# DNA Nanotechnology in the Undergraduate Laboratory: Isothermal Assembly and Quantification of DNA Nanostructures

**DOI:** 10.1021/jacsau.6c01181

**Published:** 2026-09-04

**Authors:** Hannah Talbot, Sarah Youssef, Arun Richard Chandrasekaran

**Affiliations:** † Department of Nanoscale Science and Engineering, University at Albany, State University of New York, Albany, New York 12222, United States; ‡ Department of Biological Sciences, University at Albany, State University of New York, Albany, New York 12222, United States; § The RNA Institute, University at Albany, State University of New York, Albany, New York 12222, United States

**Keywords:** Upper-division undergraduate, Biochemistry, Hands-on learning/manipulatives, Electrophoresis, Nanotechnology, Nucleic acids/DNA/RNA, Undergraduate research, DNA nanotechnology

## Abstract

DNA nanotechnology has proven to have strong potential in several applications. This technology relies on the programmable nature of DNA self-assembly to build structures and motifs with a high level of precision. Most assembly techniques rely on a temperature gradient that might not be suitable for all applications, especially those involving other biomolecules. Here, we introduce the isothermal assembly concept that relies on a single temperature to assemble DNA nanostructures. This simple concept can be easily implemented in undergraduate-level training. We present a simple experimental setup for introducing undergraduate students to DNA nanotechnology and learning characterization techniques such as gel electrophoresis. We believe that basic DNA nanotechnology protocols can boost the level of undergraduate research and enrich the interdisciplinary skill set of students, especially those working in molecular biology and biochemistry laboratories.

## Graphical abstract

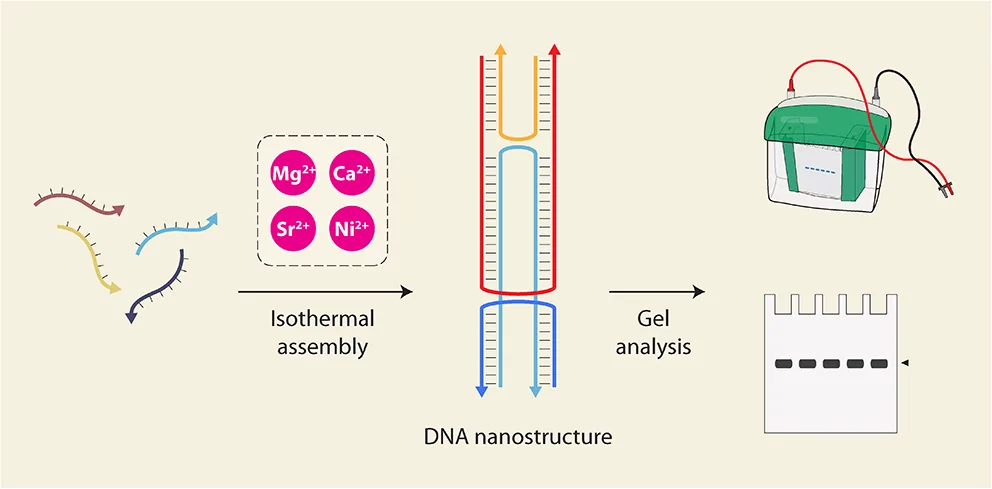

## Introduction

The use of DNA as a building block for the construction of nanoscale structures has been demonstrated in finite objects, two-dimensional arrays and three-dimensional crystals.^1–3^ Due to the programmable nature of this method that allows arbitrary designs with significant precision, DNA nanostructures have found applications in several areas including drug delivery, diagnostics, data storage, and bioimaging.^4–7^ Typically, most DNA nanostructures are assembled using a thermal annealing process where the mixture of component DNA strands and buffer is heated above the melting temperature of the DNA strands (90–95 °C) and slowly cooled to yield thermodynamically stable structures.^8–10^ Recently, work by our group and others have introduced isothermal assembly as an alternative to thermal annealing, i.e. assembling DNA nanostructures at constant moderate temperatures.^11–20^ Isothermal assembly can provide a convenient method bypassing high temperatures, allowing nanostructure preparation in low-resource settings. Assembly at constant temperatures also allows coassembly of temperature sensitive guest biomolecules. Some examples of structures made by isothermal assembly are small DNA motifs, DNA nanotweezers and DNA origami structures.^11–18^ Since DNA nanostructure design and preparation takes several steps and complexity, we selected isothermal assembly as a simple technique to introduce students to the field of DNA nanotechnology using low-cost equipment readily available in educational laboratories. We demonstrate this method in an undergraduate setting using a small DNA motif since the characterization of larger DNA nanostructures requires imaging techniques such as atomic force microscopy (AFM) and transmission electron microscopy (TEM) that also need extensive training. On the contrary, smaller DNA nanostructures are readily characterized by gel electrophoresis, an often-used method that allows inexpensive characterization and quantification of smaller DNA nanostructures rendering it more suitable for undergraduate training. Gel electrophoresis is one of the most commonly used techniques for the detection of DNA fragments and assemblies, and several laboratory experiments based on gel electrophoresis have previously been tailored for the undergraduate and high school level students.^21–25^ Further, gel-based analysis of DNA nanostructures also provides a quantitative estimate for the yield of the assembled nanostructures.^26^

DNA nanotechnology has expanded in recent years to involve several laboratories focusing on varied interdisciplinary research. While the field has shifted from assembly to applications, only recently have there been efforts to create educational and outreach aspects of the field. Key additions in this regard have been review articles on overarching topics, the development of simulation and nanostructure design techniques that cater to nonexpert scientists,^27,28^ and outreach activities on DNA nanotechnology.^29^ Laboratory exercises on the assembly and characterization of DNA nanostructures have also been developed for high school and undergraduate audiences,^25^ including our own resources on the analysis of molecular topology,^23^ biostability,^24^ and unconventional conformations^30^ of DNA nanostructures. Several other articles and laboratory protocols have introduced various concepts of biomolecular nanotechnology to students.^31–35^ The current laboratory experiment adds to this series of demonstrations of DNA nanotechnology concepts in an undergraduate laboratory setting.

In this work, we developed a protocol to isothermally assemble a model DNA nanostructure, the double crossover (DX) DNA motif, in different counterions and perform analysis and quantification by gel electrophoresis (Figure 1a). We provide both instructor and student manuals for identifying assembled DX nanostructures using native polyacrylamide gel electrophoresis (native-PAGE) with a detailed step-by-step protocol for reagent preparation and an experimental procedure. Students will mix DNA strands, assemble DX nanostructures at a constant temperature, and analyze samples by native-PAGE. These experiments are intended to introduce students to DNA nanotechnology and enhance their laboratory skills by working with a technique common in almost every biochemistry and biology lab. This protocol is suitable for third-year biology or chemistry undergraduate students, specifically those in biochemistry and genetics lab courses where students already run gels (as in our own institution). This experiment will address two teaching concepts: (1) experimental demonstration of DNA nanostructure assembly in different counterions and (2) quantification of assembly yields using gel electrophoresis. Discussions of self-assembly and nanotechnology to students, combined with experiential learning, convey scientific concepts better at the undergraduate level,^36^ translating theory-based learning with experiments^37^ and improving hands-on skills in a laboratory setting.^38,39^

**Figure 1:**
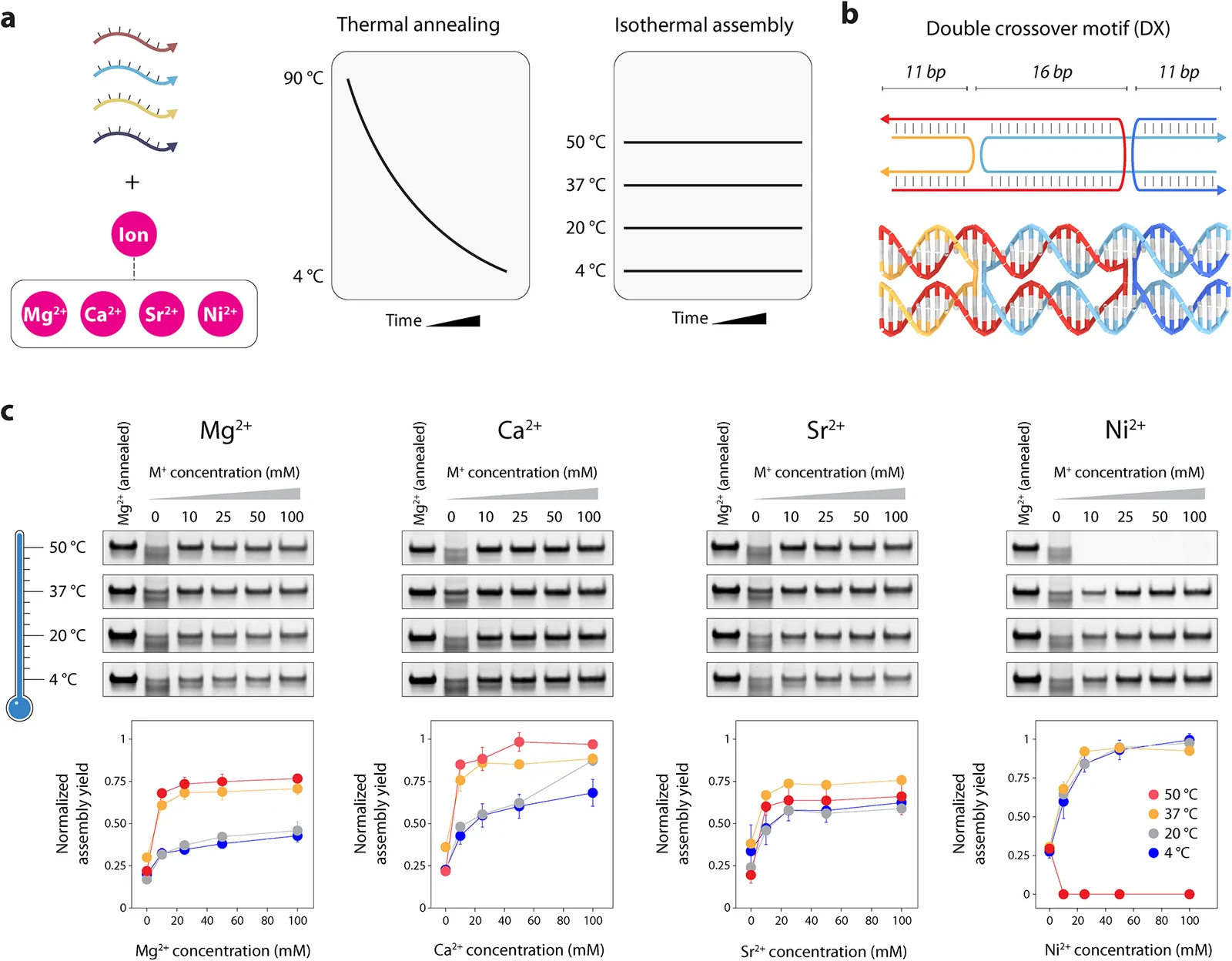
**Isothermal assembly of DNA nanostructures**. (a) Illustration comparing thermal annealing and isothermal assembly in different counterions. (b) Design of the double crossover (DX) DNA motif used as a model nanostructure. (c) Native PAGE results showing DX assembly in buffer containing counterions Mg^2+^, Ca^2+^, Sr^2+^, or Ni^2+^ at different assembly temperatures. Reproduced from ref.^11^ Available under a CC BY- NC license. Copyright 2025 Rodiguez et al.

## Hazards

Acrylamide and tetramethylethylenediamine (TEMED) are to be handled with caution. Acrylamide is carcinogenic, while TEMED is a respiratory irritant when inhaled. Gloves and protective gear should be worn during performance of gel electrophoresis experiments. Additionally, gel chambers and electrodes should be handled with caution to avoid an electric shock. Using the gel imager requires additional caution, and appropriate skin protection is recommended to avoid potential exposure to UV-irradiation.

## Concepts and Results

For this laboratory experiment, we used the DX DNA motif as the model nanostructure. The DX structure is assembled from four component single strands, forming two double helical domains connected by two crossover points. In our model structure, the crossovers are antiparallel and separated by an odd number of half turns (Figure 1b, Figure S1 and Table S1). We chose the DX nanostructure since it requires only four strands to assemble and does not rely on sophisticated imaging techniques to characterize, making it ideal for an educational lab setup. The motif is also well characterized by our group and others, and is widely used in DNA nanotechnology in the creation of multihelical objects and 2D arrays,^40,41^ with applications in molecular computation, catalysis and biomolecular scaffolding.^42–44^ In our prior work, we demonstrated the assembly of the DX and other DNA motifs in different counterions instead of the typically used Mg^2+^.^45^ Further, we also developed an isothermal assembly method for a variety of DNA nanostructures in different counterions,^11^ showing efficient assembly at constant temperatures of 4, 20, 37, and 50 °C. Notably, while divalent ions such as Mg^2+^, Ca^2+^ and Sr^2+^ enabled proper assembly at all the temperatures tested, Ni^2+^ was sensitive to temperature, with DNA nanostructures assembling in Ni^2+^ only at temperatures below 40 °C (Figure 1c). The different effects of counterions, combined with assembly of DNA nanostructures at room temperature, makes this experiment an ideal fit for an undergraduate lab requiring only basic equipment commonly used in most educational laboratories. Our previous results showed that most ions examined had the highest yields of correctly folded structures at concentrations above 50 mM and temperatures starting from 37 °C. We observed an ion concentration dependent assembly yield increase at 20 °C. Considering these prior results, we chose 20 and 50 °C as the isothermal assembly temperatures for this undergraduate laboratory experiment, in counterions Mg^2+^, Ca^2+^, Sr^2+^ and Ni^2+^. These conditions allow the students to compare the effect of temperature on isothermal assembly of DNA nanostructures. This effect will be especially noticeable in Ni^2+^ since it previously showed significantly poor yield than other ions at 50 °C compared to 20 °C.

In the educational experiment we performed in our laboratory, students were provided with DNA strands and conducted the experimental protocol adjusted to fit settings and time frame of typical undergraduate laboratory sessions (∼3 h) (Figure 2). To enable educational institutions to adapt our protocol, we have created a detailed protocol for sample preparation by instructors (Supplementary Note 1). For students to perform the experiment, we also provide a detailed manual with a step-by-step protocol for DNA nanostructure assembly, gel electrophoresis, and gel imaging and analysis (Supplementary Note 2). Aliquots of reagents and buffers were provided to students at ready-to-use concentrations (Table S2).

**Figure 2:**
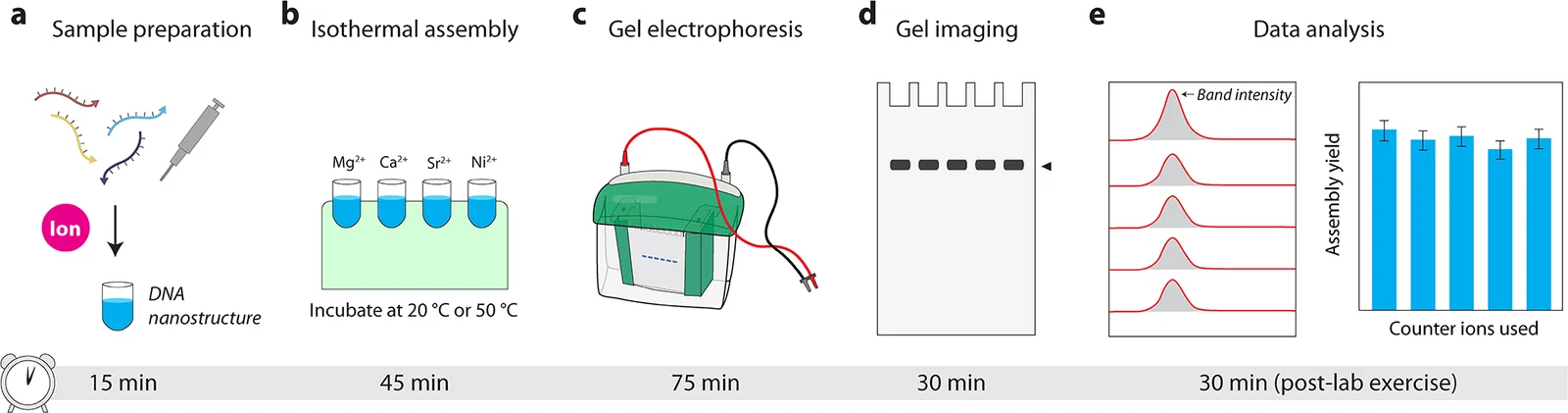
**Workflow of isothermal assembly of DX DNA motif in different ions at 20 or 50 °C**. Estimated timing for each step is shown below the experimental steps: (a) Mixing component DNA strands with buffer and ion solution, (b) incubating two sets of samples, one at 20 °C and one at 50 °C, (c) Samples are run on native-PAGE, (d) Gel is stained with GelRed and imaged, (e) Band intensities are quantified and plotted against temperature for each ion.

First, the students mixed the strands at equimolar ratios, then incubated the samples at 20 or 50 °C for 45 min. Next, students ran native-PAGE experiments for DX structures isothermally assembled in 100 mM concentration of different ions Mg^2+^, Ca^2+^, Sr^2+^ and Ni^2+^. All the samples were prepared in the commonly used Tris-acetate-EDTA buffer (1× TAE, pH 7.8) supplemented with Mg^2+^, Ca^2+^, Sr^2+^ or Ni^2+^. The assembly of DX was confirmed by comparing the gel bands of isothermally assembled structures with those annealed by a thermal gradient in 12.5 mM Mg^2+^ (provided to the student). Assembly yields were quantified using Image Lab gel analysis software and normalized to the thermally annealed control samples. The gels were performed by three different students, and the data was compared to previous gels performed earlier in our lab. The samples were run on 8% native-PAGE at 20 °C for 45 min. Following electrophoresis, gels were stained with the nontoxic dye GelRed and imaged on BioRad GelDoc imager. Figure 3 shows the representative gel images from experiments performed by three different students. Students observed the isothermal assembly of the DX DNA motif at 20 and 50 °C, with a predominant single band migrating at the same level as the control annealed sample in Mg^2+^. Students’ gel data also showed proper assembly in Ni^2+^-containing buffer only at 20 °C and not at 50 °C (Figure 3).

**Figure 3:**
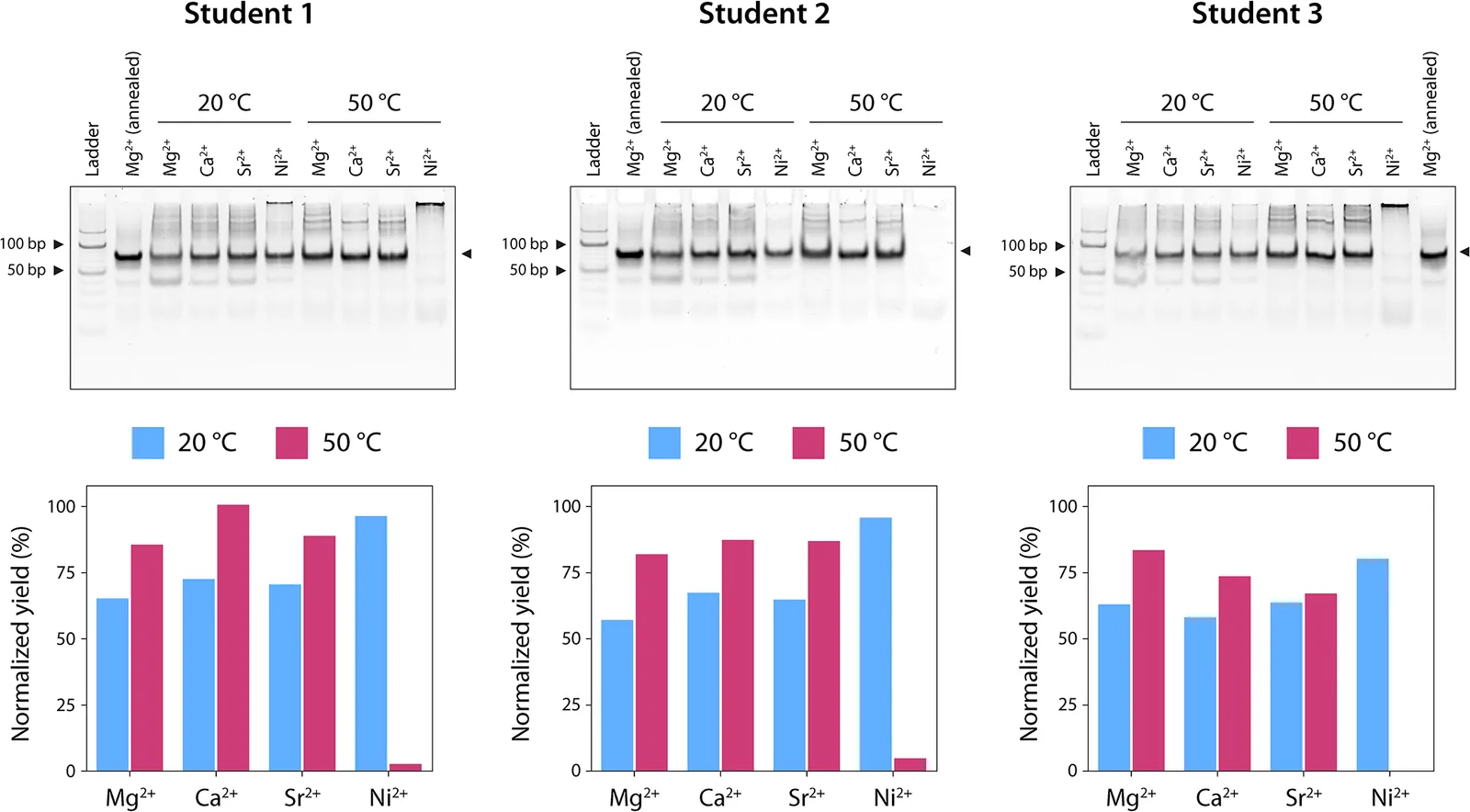
**Representative results for isothermal assembly and quantification performed by three undergraduate students**. Students were given the manual in Supplementary Note 2 and were provided with annealed DNA controls and reagents. Gel analysis was done using ImageLab and data plotted using Origin. Each student performed isothermal assembly at both 20 and 50 °C for comparison.

## Discussion

The educational experiment presented here aims to introduce students to basic principles of DNA nanotechnology through isothermal assembly of a model DNA nanostructure in several ions and at different temperatures. The total duration of experiment was planned to be ∼ 3 h to provide students sufficient time to complete the experiment in a typical lab course duration. While we allotted specific timings for each step, some of the steps can overlap and can be performed in parallel to save time. For example, our protocol indicates 60 min for sample preparation (15 min to mix and 45 min isothermal incubation) and a separate 75 min duration for gel preparation and running. Instructors can guide the students to first prepare the gel and then start the sample preparation while the gel is polymerizing. We had first considered the gel analysis to be part of the in-lab exercise. However, with several trials by undergraduate students, it did not seem feasible within a 3-h duration. The gel analysis is also suitable to be a postlab exercise, and students can be instructed to perform it remotely (Supplementary Note 3). In our lab demonstration, all the students were able to perform lab experiments within the suggested time frame. We also provide a troubleshooting guide for instructors and students to avoid potential mistakes that can cost material and time (Supplementary Note 4). The experimental results slightly varied between the three students, and differences in assembly yields with the published results could arise from pipetting errors during mixing and gel loading, but the overall results follow similar trends (Figure S2). Thus, multiple students can also be grouped together in a lab setting to perform replicate experiments. Instructors can also modify the experiment to include stepwise assembly of the DX motif to discuss partial assembly of DNA nanostructures with students (Figure S3 and Supplementary Note 5).

Our laboratory exercise already has some variations from our published work to make it suitable for an undergraduate setting. Two key aspects are (1) gels are run at room temperature for this exercise instead of cold room so that any institution can easily adapt this protocol, and (2) shorter incubation times at higher ion concentrations allow instructors to tailor the protocol to be performed with 2.5–3 h. This experimental setup is suitable for early stage undergraduates with some lab experience or third- and fourth-year undergraduates for whom this is similar to a typical biochemistry or molecular biology lab experiment involving pipetting and gel running. Because the experiment does not require any thermal cyclers or expensive imaging microscopes, the typical cost is minimal. The cost of strands is ∼ $50 for all four strands together (each at the 100 nmole scale). Purchased strands are usually reconstituted to 100 μM which would last for several experiments across several years (we suggest 10 μL of 0.5 μM sample per lane). The experimental setup is typically available in most biochemistry and molecular biology laboratories. For institutions that do not have easy access to a standard benchtop gel imager, alternative strategies that use camera-based gel imaging and visible dyes could be used.^46,47^ A typical gel chamber can run four gels simultaneously; thus, a lab section with 20–30 students would require 5–6 gel electrophoresis set ups. Further, recent developments in 3D-printed gel apparatus could be adopted to offset initial costs related to gel set ups.^48^ Specifically, the GelRed stain we use is a suitable nontoxic alternate to the toxic ethidium bromide that requires additional waste disposal measures or the more expensive SYBR gold stain.

Overall, our protocol adds to the very few existing DNA nanotechnology educational articles, with a focus on introducing students to isothermal assembly of DNA nanostructures and the effect of different counterions. The workflow is also highly adaptable in an undergraduate setting, including Primarily Undergraduate Institutions (PUIs), as it uses routine equipment and reagents. Our protocol is also aimed at improving hands-on laboratory skills for students by performing one of the most commonly used techniques of gel electrophoresis in biology and chemistry laboratories. Additionally, the pedagogical aspect also includes data analysis, allowing students to reflect on their results compared with published data. For our lab, this experiment exposed students to a cutting-edge research topic, established research techniques including data analysis, and presented such results in our progress assessment meetings. The isothermal DNA assembly technique offers a unique and feasible setting for students to get introduced to DNA nanotechnology, enhancing their skillset for a variety of interdisciplinary fields of research.

## Supplementary Material



## Notes

Materials and methods, notes for instructors, student instruction manual, worksheet for students, troubleshooting guide and gel analysis instructions (PDF)
