# Supplementary material for: DNA Nanotechnology in the Undergraduate Laboratory: Isothermal Assembly and Quantification of DNA Nanostructures
Source: JACS Au. 2026 Sep 4;6(9):5510–5. doi: 10.1021/jacsau.6c01181 (PMC13625532; doi:10.1021/jacsau.6c01181)
Supplement: Supplementary file 1 [file au6c01181_si_001.pdf]

# DNA nanotechnology in the undergraduate laboratory: Isothermal assembly and quantification of DNA nanostructures

Hannah Talbot,[1,2] Sarah Youssef,[1] and Arun Richard Chandrasekaran[1,3,*]

[1]Department of Nanoscale Science and Engineering, University at Albany, State University of New York, Albany, NY 12222, USA.
[2]Department of Biological Sciences, University at Albany, State University of New York, Albany, NY 12222, USA.
[3]The RNA Institute, University at Albany, State University of New York, Albany, NY 12222, USA.

*Correspondence: arun@albany.edu
[#] Joint first authors

## Contents

- General methodology
- Additional experimental results
- Supplementary note 1: Reagent preparation for instructor
- Supplementary note 2: Student instruction manual
- Example student worksheet and post-lab questions
- Supplementary note 3: Gel analysis instructions
- Supplementary note 4: Trouble shooting guide
- Supplementary note 5: Stepwise assembly of DX DNA motif

## General methodology

*Oligonucleotides*

All DNA strands were purchased from Integrated DNA technologies (IDT) and reconstituted in nuclease free water to a stock concentration of ~100 µM. DNA strand concentrations were calculated based on absorbance measured using a Nanodrop 2000 spectrophotometer and further dilutions were made in nuclease free water. Sequences of the DNA strands used are shown in **Table S1**.

*Calculating DNA concentration*

The concentration of DNA is determined using a UV spectrophotometer, or a NanoDrop, a spectrophotometer where only a few microliters of the sample is required for measurement. Deionized water was used as the blank for the measurements. A small amount of sample (typically 1-2 µl) was pipetted, and the absorbance at 260 nm was recorded. The measurements were repeated 3 times, and the average of the absorbance was used to determine concentration using Beer lambert's equation:

$$A = \varepsilon bc$$

Where:
A = absorbance at 260 nm
$\varepsilon$ = molar extinction coefficient of DNA strand in $L.mol^{-1}.cm^{-1}$ (provided in vendor datasheet)
b = path length (typically b= 1 cm)
c = concentration in mol/L

*Preparation of counter ion solutions*

For different metal ions, the chemicals used were magnesium acetate tetrahydrate, calcium chloride dihydrate, strontium chloride hexahydrate, and nickel (II) chloride hexahydrate. 1 M stock solutions of these salts were prepared in de-ionized water and used in the experiments.

*Assembly of the DNA double crossover (DX) motif*

DNA strands were diluted to 5 µM intermediate stocks and mixed at equimolar concentrations yielding a final concentration of 0.5 µM. DNA strands were mixed with tris-acetate-buffer (TAE) buffer (40 mM Tris base (pH 8.0), 20 mM acetic acid, 2 mM EDTA, 1× final) with different ions at a final concentration of 100 mM for each ion. Step by step instructions for the lab instructor are provided in Supplementary Note 1.

*Polyacrylamide gel electrophoresis*

Native polyacrylamide gels (8%) were made in 1× TAE buffer containing 12.5 mM $Mg^{2+}$ and gels were run at 100 V (constant voltage) for 45 minutes at room temperature. In our lab, we use the Mini-PROTEAN Tetra Vertical Electrophoresis system (BioRad). Different electrophoresis systems may require voltage and running time optimization. The gels were removed from the electrophoresis chamber and stained with 1× Gel Red (5 µl stock 10,000× Gel Red mixed into 50 ml water) for 10 min followed by destaining in water for another 5 min. Gels were then imaged using a Bio-Rad Gel Doc XR+ imager using the default settings for GelRed with UV illumination.

*Gel image analysis and quantification*

Images were quantified using Image Lab software provided with the imager (free to download online). The exposure time was selected based on the highest exposure that provides bands without saturated pixels. The selected band intensity was recorded and the data plotted to compare the folding yields among different ions. Data were normalized to the band intensity corresponding to the sample annealed in $Mg^{2+}$-containing buffer and plotted using Origin Pro software.

**Figure S1.** Design and sequence of DX DNA motif.

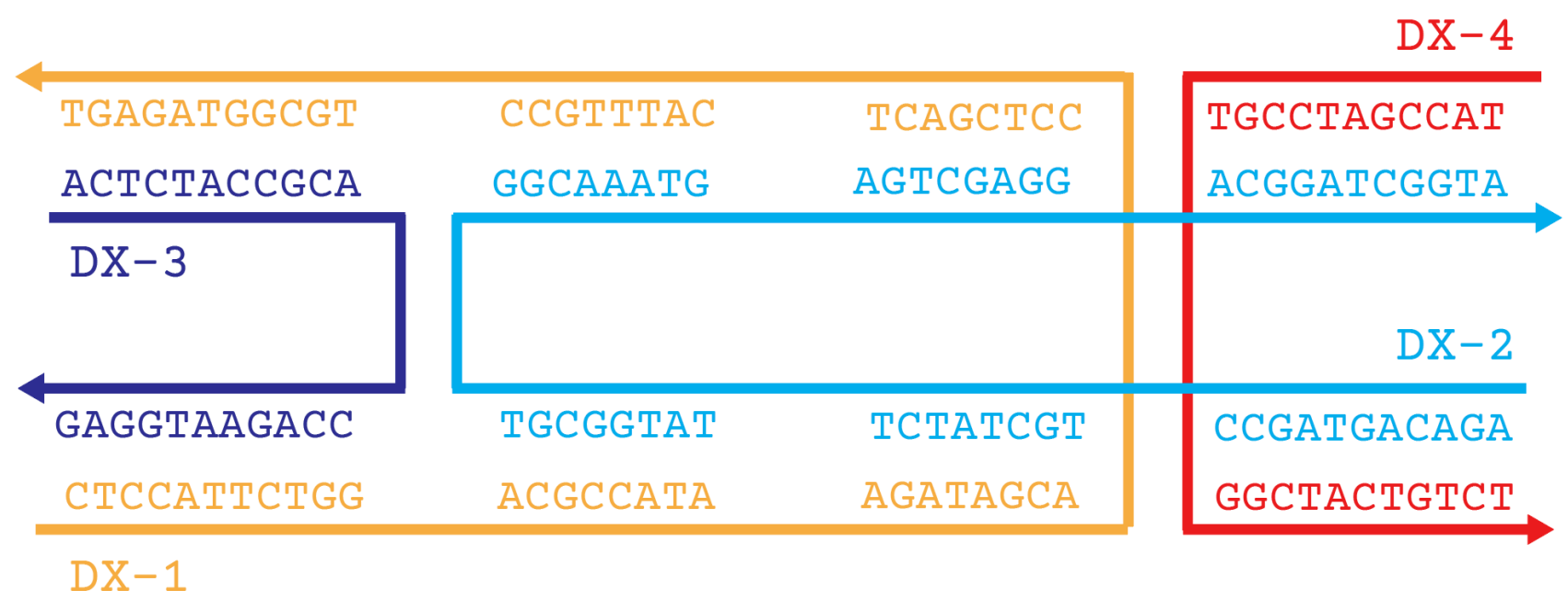


**Figure S2.** Comparison of data from three students.

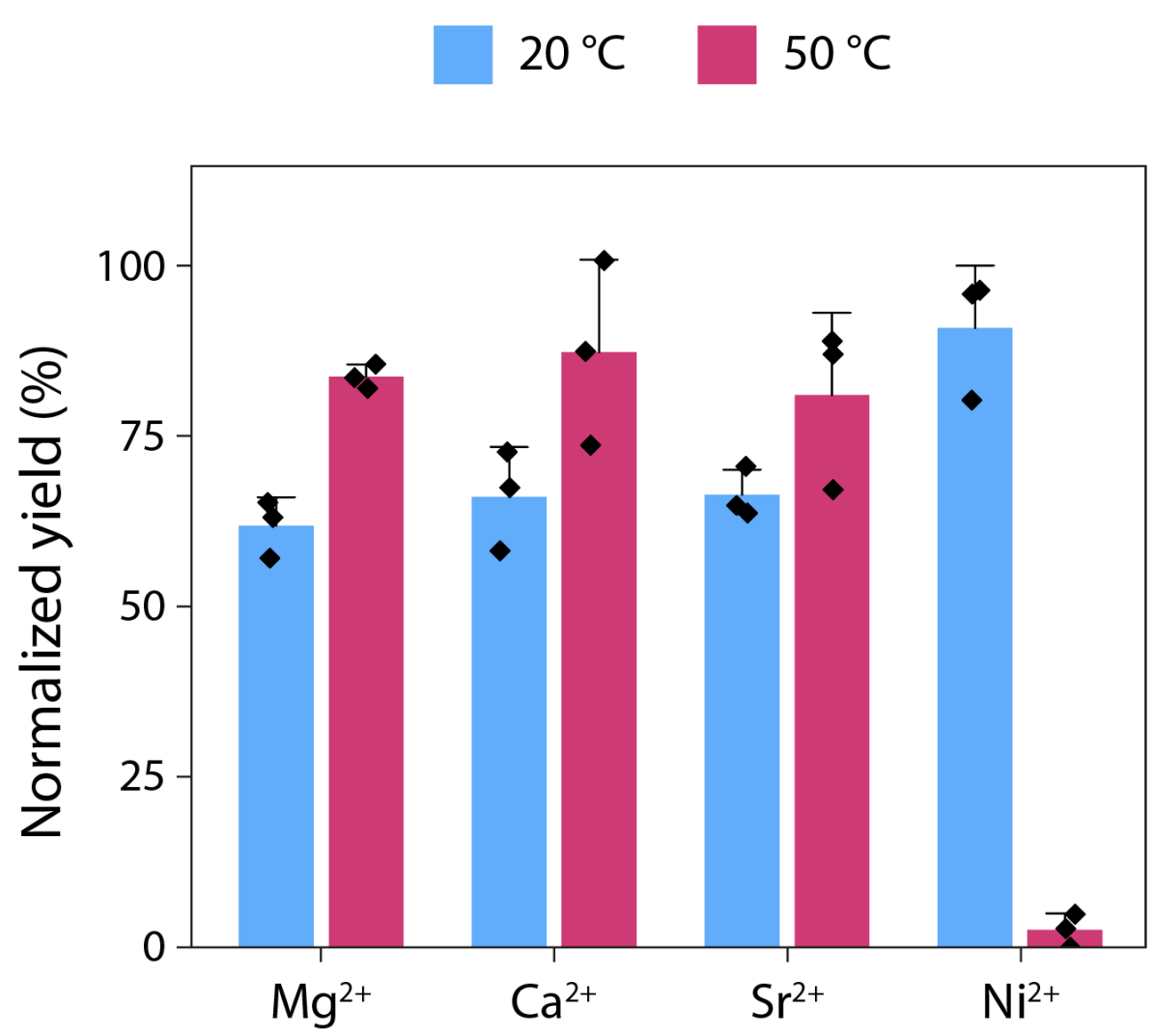


**Figure S3.** Isothermal stepwise assembly of DX DNA motif.

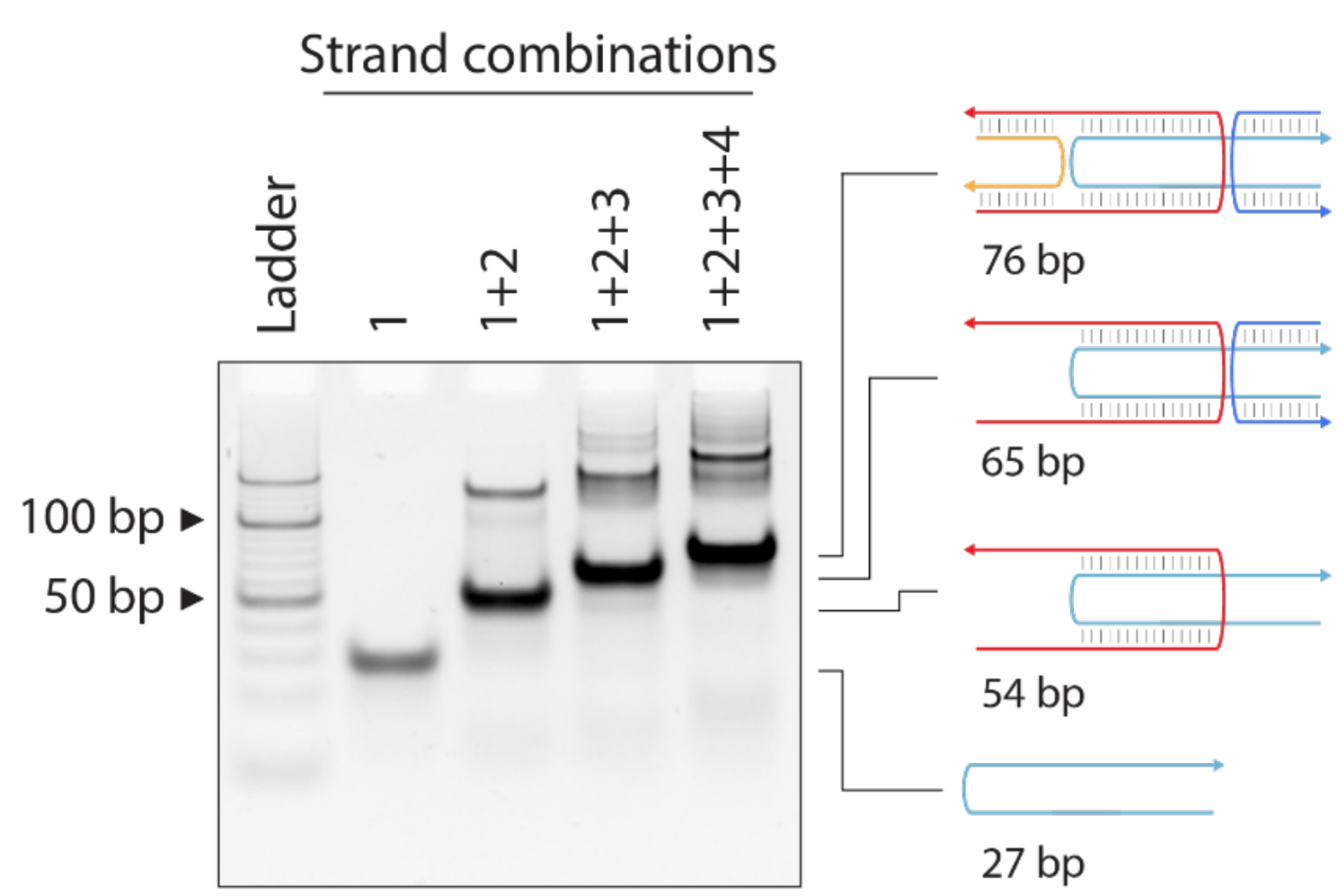

**Table S1.** Sequences of DNA strands used in the experiment.

| Strand | Sequence (5′-3′) |
|---|---|
| DX1 | CTCCATTCTGGACGCCATAAGATAGCACCTCGACTCATTTGCCTGCGGTAGAGT |
| DX2 | AGACAGTAGCCTGCTATCTTATGGCGTGGCAAATGAGTCGAGGACGGATCGGTA |
| DX3 | ACTCTACCGCACCAGAATGGAG |
| DX4 | TACCGATCCGTGGCTACTGTCT |

**Table S2.** List of reagents used in this experiment.

| Reagent | Supplier | Catalog # |
|---|---|---|
| Nuclease-free water | Invitrogen | 10977-015 |
| 40% polyacrylamide solution (19:1 acrylamide/bisacrylamide) | National Diagnostics | EC-850 |
| GelRed (10,000× in water) | Biotium | 41003 |
| Ammonium persulfate (APS) | Fisher Scientific | BP179-100 |
| Tetramethylethylenediamine (TEMED) | Thermo Scientific | PI17919 |
| 10 bp DNA ladder | Thermo Fisher Scientific | SM1313 |
| Bromophenol blue | Fisher Scientific | AAA1846918 |
| Glycerol | Hampton Research | HR2-623 |
| Tris base | VWR | 97061-794 |
| Acetic acid (1 M solution) | Fisher Scientific | S25840A |
| EDTA (0.5 M solution, pH 8) | VWR | BDH7830-1 |
| Magnesium acetate tetrahydrate | Sigma Aldrich | BP215-500 |
| Calcium chloride dihydrate | Sigma Aldrich | 21102 |
| Strontium chloride hexahydrate | Thermo Scientific | 033393-22 |
| Nickel (II) chloride hexahydrate | Sigma Aldrich | 223387 |

# Supplementary note 1: Reagent preparation for instructor

### Apparatus required

- Weighing scale
- 2 liter beaker
- Hotplate with stirrer
- Magnetic stirrer
- 1 liter storage bottles
- 2 µl, 10 µl, 20 µl, 100 µl and 1000 µl pipette and corresponding pipette tips
- PCR tubes
- 0.5 ml tubes
- Tray to hold tubes
- 15 ml and 50 ml falcon tubes
- Thermal cycler
- Gel electrophoresis plates, chamber and comb (Mini-PROTEAN Tetra Vertical Electrophoresis Cell or similar)
- Power supply for gel electrophoresis (BioRad PowerPac Basic Power Supply or similar)
- GelDoc imaging station or similar (Bio-Rad Gel Doc XR+ imager)
- Gel analysis software such as Image Lab, Image J or similar
- Graphing software (Origin Lab or similar)

Optional materials
- Gel loading tips
- Heating block (set at 50°C)
- Styrofoam box
- Sample floater
- Parafilm
- Microwave

### Chemicals and reagents required

- Oligonucleotides listed in Table S1.
- Reagents listed in Table S2.

### Hazards

Acrylamide is a hazardous chemical and carcinogen, and exposure can occur via inhalation (if aerosolized), ingestion, and skin absorption. While toxicity and potential for exposure substantially decrease after polymerization, care should be taken when using polyacrylamide to prepare gels. TEMED is respiratory irritant when inhaled. Gloves and protective gear should be worn when performing gel electrophoresis experiments. Electrocution is also a potential hazard as typical voltages of 100 V can be applied across gels. To avoid electric shocks, students should use care when plugging the gel boxes into the power supply.

## Preparation of reagents and solutions

Tris-Acetate-EDTA with magnesium (10× TAE-$Mg^{2+}$), 1 liter

*Contains 400 mM Tris base (pH 8.0), 200 mM acetic acid, 20 mM EDTA, and 125 mM magnesium acetate.*
48.5 g Tris base
200 ml acetic acid (1 M solution)
40 ml EDTA (0.5 M solution)
26.8 g magnesium acetate tetrahydrate
Make up to 1 liter using de-ionized water
Store up to 1 year at room temperature

Tris-Acetate-EDTA without $Mg^{2+}$ (10× TAE), 100 ml

*Contains 400 mM Tris base (pH 8.0), 200 mM acetic acid, and 20 mM EDTA*
4.85 g Tris base
20 ml acetic acid (1 M solution)
4 ml EDTA (0.5 M solution)
Make up to 100 ml using de-ionized water
Store up to 1 year at room temperature

Tris-Acetate-EDTA with magnesium (1× TAE-$Mg^{2+}$), 1 liter

100 ml 10× TAE-$Mg^{2+}$ (see above recipe)
900 ml de-ionized water
Store up to 1 year at room temperature

10% ammonium persulfate (APS), 5 ml

0.5 g ammonium persulfate in 5 ml of de-ionized water (prepare fresh)

10 bp ladder (preparation for 1 lane, 10 µl provided for each student)

0.5 µl 10 bp ladder
1 µl 10× TAE-$Mg^{2+}$
8.5 µl de-ionized water

Gel loading dye, 1 ml

500 µl glycerol
100 µl 10× TAE-$Mg^{2+}$ (see recipe)
400 µl deionized water
Bromophenol blue (add for visible color)

Counter ion solutions

We prepared magnesium acetate tetrahydrate, calcium chloride dihydrate, strontium chloride hexahydrate, and nickel (II) hexahydrate solutions at 1 M for use at a final concentration of 100 mM for DNA sample preparation.

**Thermal annealing of DX DNA nanostructures**

The DNA solution is annealed in a thermal cycler with the following steps: 90 °C for 3 minutes, 65 °C for 20 minutes, 45 °C for 20 minutes, 37 °C for 30 minutes, 20 °C for 30 minutes, and the solution was then cooled to 4 °C. Alternatively, the DNA solution can be slowly cooled from 95 °C to 20 °C by placing the tubes in 1 liter of hot water in a Styrofoam box for 24 hours to facilitate hybridization. Once annealed, the samples can be stored in the refrigerator (4 °C).

a. Make 5 µM dilutions for the DNA strands listed in Table S1.

b. Mix the component strands in equimolar ratios (using the 5 µM dilutions) to a final concentration of 0.5 µM in 1× TAE/$Mg^{2+}$ buffer (volumes shown below are for preparing 200 µl). The volume to be made depends on the number of students performing the experiment. Each student will need 10 µl of the annealed sample.

| | |
|---|---|
| Strand DX1 (5 µM) | 20 µl |
| Strand DX1 (5 µM) | 20 µl |
| Strand DX1 (5 µM) | 20 µl |
| Strand DX1 (5 µM) | 20 µl |
| 10× TAE/$Mg^{2+}$ buffer | 20 µl |
| Deionized water | 100 µl |

<u>For thermal cycler:</u> Samples can be prepared in a 0.2 ml PCR tube if using a thermal cycler. Set up the annealing protocol and once the lid temperature reaches the maximum temperature, place the sample tubes in the thermal cycler.

<u>For water bath:</u> If annealing in a water bath, use a 0.5 ml or 1 ml tube, close the tube and use parafilm to seal the tube lid to prevent opening of the tube lid and evaporation of the sample. Heat 1 liter of water in a 1-liter beaker using a hot plate or a microwave until the temperature reaches 90 °C. Place the 1-liter beaker containing heated water in a Styrofoam box. Make sure that the Styrofoam box size fits the 1-liter beaker and allows it to be closed fully. Place the tube containing the DNA mixture on a floater and place the floater on the water in the beaker. Close the Styrofoam box and tape it and leave it undisturbed for 24 h.

**Supply checklist for each student/group**

*Some volumes have been doubled for easy pipetting; this can be adjusted to what materials are available.*

**Sample preparation**

5 µl 1 M magnesium acetate tetrahydrate solution
5 µl 1 M nickel (II) chloride hexahydrate solution
5 µl 1 M strontium chloride hexahydrate solution
5 µl 1 M calcium chloride dihydrate solution
20 µl 5 µM DX1
20 µl 5 µM DX2
20 µl 5 µM DX3
20 µl 5 µM DX4
20 µl 10× TAE
80 µl deionized water
20 PCR tubes

**Gel**

15 ml or 50 ml conical tube
4 ml 40% polyacrylamide gel solution
2 ml 10× TAE with $Mg^{2+}$
14 ml deionized water
8 µL TEMED
160 µl 10% APS
10 µl annealed DX control
10 µl 10bp ladder
20 µl PAGE loading dye
10 µl GelRed

**Shared tools**

2 µl, 10 µl and 100 µl pipettes and the appropriate pipette tips
Serological pipette
Thermal cycler or heating block
Gel set up
Power supply
Graduated cylinder
Staining tray
Shaker or rotator
Aluminum foil
Gel imager and gel imaging software

## Supplementary note 2: Student instruction manual

DNA nanotechnology aims to build bottom-up DNA based nanostructures for applications such as drug delivery, biosensing applications and data storage. The advantage of such structures is the ability to include other functionalities with a high degree of precision in terms of space and number. Traditional DNA nanostructure assembly involves the heating of component DNA mixtures to a high temperature (typically 90-95 °C) and slow cooling to room temperature (~20 °C) or 4 °C to fold the nanostructures into predesigned shapes. However, this method might not be optimal when including other biomolecules (eg: proteins) as guests. To solve this problem, isothermal assembly, especially at room temperature, offers a potential solution. In this experiment, we will examine the isothermal assembly of DNA nanostructures using a model DNA nanostructure called the double crossover (DX) motif and analyze the effect of different counterions in the solution on the assembly yield.

To learn more about DNA nanotechnology, you can watch this video of a seminar by Prof. Nadrian Seeman, founder of the field of DNA nanotechnology:
https://www.youtube.com/watch?v=Dh0v7clN0yM.

**Apparatus required**

- 2 µl, 10 µl and 100 µl pipette and pipette tips
- 10 ml serological pipets and pipette tips
- Gel electrophoresis set up
- Gel power supply
- Gel imager
- Shaker
- Aluminum foil
- Thermal cycler/heating block
- 15 ml or 50 ml conical tube
- PCR tubes
- Gel staining trays
- Graduated cylinder

**Chemicals and reagents required**

- Thermally annealed DX DNA motif
- DNA strands
- Ion solutions
- 10× TAE
- 1× TAE/$Mg^{2+}$
- GelRed
- Loading dye
- 40% polyacrylamide solution
- 10% ammonium persulfate (APS)
- Tetramethylethylenediamine (TEMED)
- Deionized water

**Hazards**

Acrylamide is a hazardous chemical and carcinogen, and exposure can occur via inhalation (if aerosolized), ingestion, and skin absorption. While toxicity and potential for exposure substantially decrease after polymerization, care should be taken when using polyacrylamide to prepare gels. TEMED is respiratory irritant when inhaled. Gloves and protective gear should be worn when performing gel electrophoresis experiments. Electrocution is also a potential hazard as typical voltages of 100 V can be applied across gels. To avoid electric shocks, students should use care when plugging the gel boxes into the power supply.

**Procedure**

Follow the below procedure step-by-step to prepare the DNA double crossover (DX) motif and analyze the assembled structures using gel electrophoresis.

### 1. Assembly of DX DNA motif in ion solutions [Timing: 60 min]

a. Mix DNA samples with buffer and ion stock solutions following the table below. The samples are mixed in PCR tubes (or 0.5 ml or 1 ml tubes). Final sample volume is 25 µl.

| **Reagent** | **Starting concentration** | **Final concentration** | **Volume required** |
|---|---|---|---|
| Strand DX1 | 5 µM | 0.5 µM | 2.5 µl |
| Strand DX2 | 5 µM | 0.5 µM | 2.5 µl |
| Strand DX3 | 5 µM | 0.5 µM | 2.5 µl |
| Strand DX4 | 5 µM | 0.5 µM | 2.5 µl |
| TAE | 10× | 1× | 2.5 µl |
| Ion solution | 1000 mM (1 M) | 100 mM | 2.5 µl |
| De-ionized water | | | 10 µl |
| Total volume | | | 25 µl |

b. Split each sample into two new tubes with 10 µl in each tube, one for incubation at room temperature and one for incubation at 50 °C.

c. Incubate the samples for 45 minutes at 20 °C and 50 °C in a thermal cycler to maintain temperature throughout the experiment.

Alternate: Mix samples in 0.5 ml or 1 ml tubes and keep on heating blocks set to 20 °C and 50 °C.

### 2. Preparing native polyacrylamide gel [Timing: 15 min]

a. Set up the gel plates and chamber according to instructor's directions.

- Mix the following to make an 8% polyacrylamide gel.

  2 ml 40% polyacrylamide
  1 ml 10× TAE/$Mg^{2+}$
  7 ml de-ionized water

  Note: Add the APS and TEMED just before the gel is ready to be poured into the gel plate set-up.

  80 μl 10% APS
  4 μl TEMED

  - Pour the gel solution in the glass plate set up using a 10 ml serological pipet. Place the comb and allow gels to polymerize for 30-40 minutes.

  - Set up gel chamber and fill with 1× TAE-$Mg^{2+}$ for gel running.

b. Rinse wells by flushing running buffer using a 200 μl pipette.

### 3. Gel electrophoresis of DX samples [Timing: 60 min]

a. Add 1 μl loading dye to all the 10 sample tubes.

b. Starting with the ladder provided by the instructor, load 10 μl of the ladder, control, and each of the 8 samples into the gel, totaling 10 lanes.

   Note: Follow this loading order for the samples, from left to right.

   Ladder
   Annealed control
   20 °C, Magnesium
   20 °C, Calcium
   20 °C, Strontium
   20 °C, Nickel
   50 °C, Magnesium
   50 °C, Calcium
   50 °C, Strontium
   50 °C, Nickel

c. Run the gel by applying 100 V for 45 minutes at room temperature.

   Note: Make sure to plug in the electrode to the matching sockets (red to red and black to black)

### 4. Gel staining and imaging [Timing: 30 min]

a. Prepare gel staining trays with 50 ml of water and add 5 μl of GelRed stock solution to the water and stir well using the pipette tip.

b. Open the gel plates and place the gel carefully in the staining solution. For convenience, gel can remain on one of the plates so that it is easier to move the gel.

c. Place the gel-containing trays on a rotator or a shaker, cover it with aluminum foil and let it stain for 10 minutes.

d. After staining, move the gel to a tray with 50 ml water. De-stain for 5 minutes.

e. Remove the gel from the de-staining tray and place on the imaging platform (without the gel plate) on a gel imager. Image using the gel analysis software. Make sure to take gel images at an exposure time at which the band corresponding to the structure is not saturated. If saturated, the intensity values will not be accurate. The exposure time in the imager can be reduced to obtain gel images without saturation of the bands.

## 5. Post-lab exercise: Gel image analysis and plotting the assembly yield

a. Use Image Lab or the image analysis software provided with the gel imaging station (or Image J) to analyze the gel. Measure the intensity of the band corresponding to the structure in the lane for each ion and incubation temperature.

b. Normalize the band intensity of each sample to the band intensity of the DX DNA in the control lane. The reduction in band intensity of each sample relative to the annealed control provides the fraction of yield of each of the metal ions at each incubation temperature.

c. Plot the normalized band intensities using Origin Lab or other similar software against temperature for each ion. An example graph is shown below.

$$Normalized\ yield = \frac{Band\ intensity\ in\ ions}{Band\ intensity\ of control} x100$$

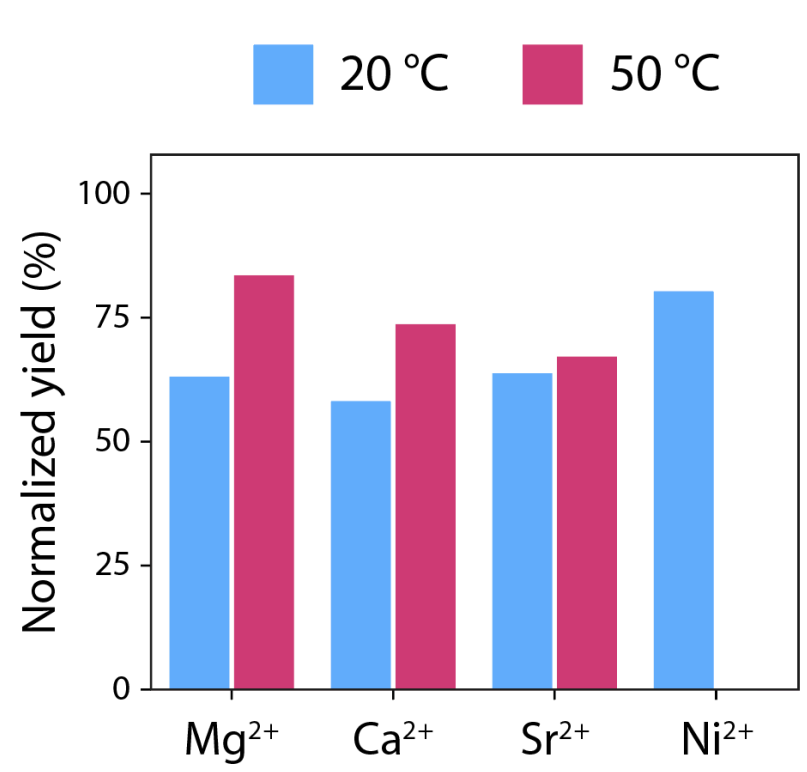

# Worksheet

1. Place your gel image here.

2. Band intensity analysis.

| Temperature | Gel lane | Band intensity | |
|---|---|---|---|
| | Control | Raw data | Normalized to control |
| 20 °C | $Ca^{2+}$ | | |
| | $Mg^{2+}$ | | |
| | $Sr^{2+}$ | | |
| | $Ni^{2+}$ | | |
| 50 °C | $Ca^{2+}$ | | |
| | $Mg^{2+}$ | | |
| | $Sr^{2+}$ | | |
| | $Ni^{2+}$ | | |

3. Print and attach graphs below.

## Post-lab questions

1. Describe the reaction mechanism for polyacrylamide gel formation.

2. Describe the differences between non-denaturing (native) and denaturing PAGE and application of each type of gel. Explain why native PAGE was chosen for DNA assembly validation.

3. What is the mechanism by which GelRed dye stains DNA?

4. What is the wavelength at which the gel is imaged using the gel imager?

5. If you have DNA structures of 25, 75 and 100 base pairs, which of these structures will migrate faster in the gel? Justify your answer.

6. What are some of the advantages of isothermal assembly of DNA nanostructures?

7. Why was no assembly observed in the buffer containing nickel?

## Supplementary note 3: Gel analysis

### 1. Quantifying band intensities from gel images

a. Open your gel file using ImageLab.

b. Click on the 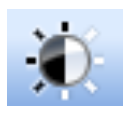 icon. Ensure "Highlight saturated pixels" is checked.

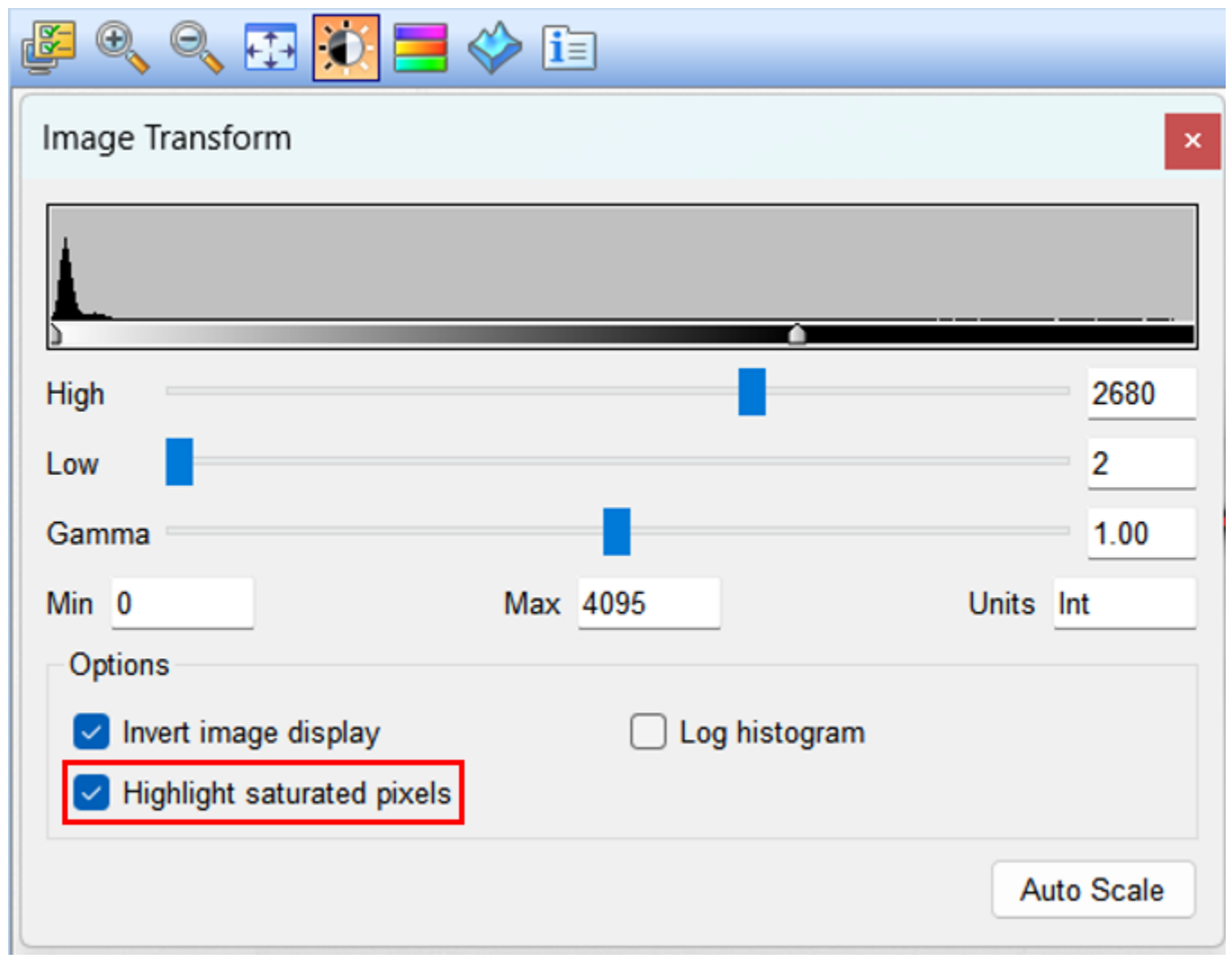


c. Check your gel image. If any red pixels are present, the gel band is oversaturated. Use a gel image with a lower exposure time that does not have any oversaturated pixels for gel analysis.

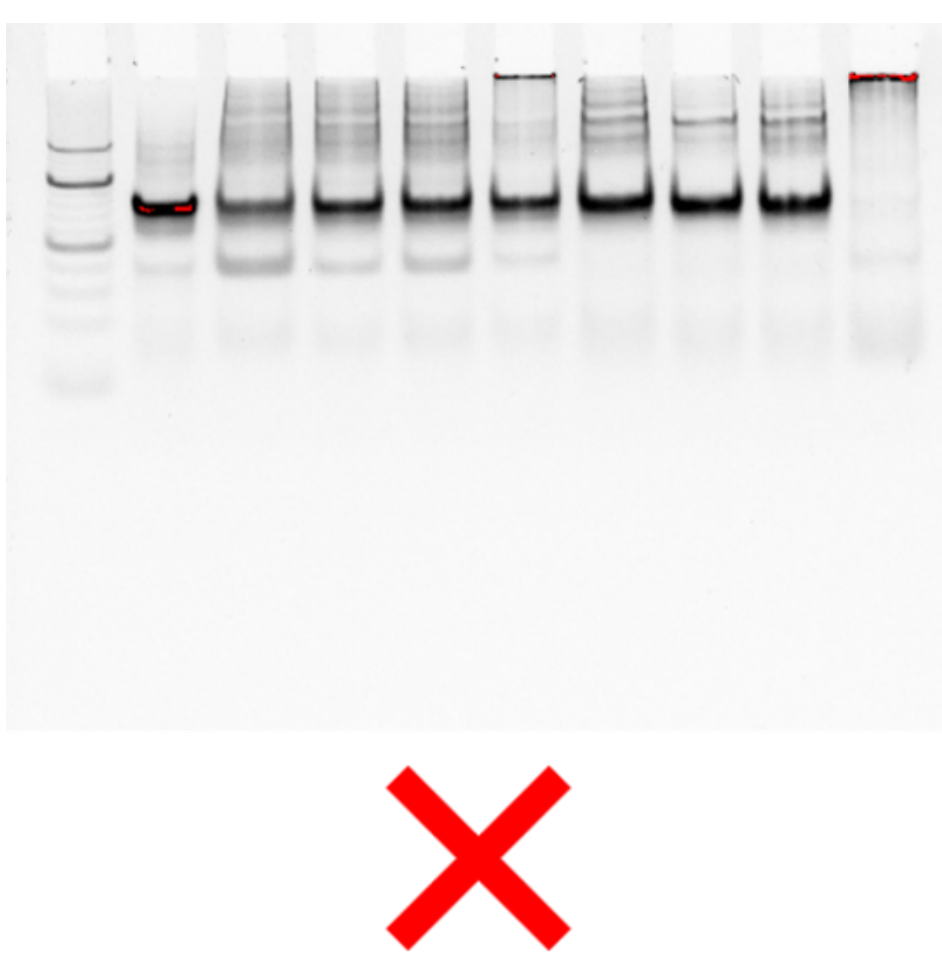

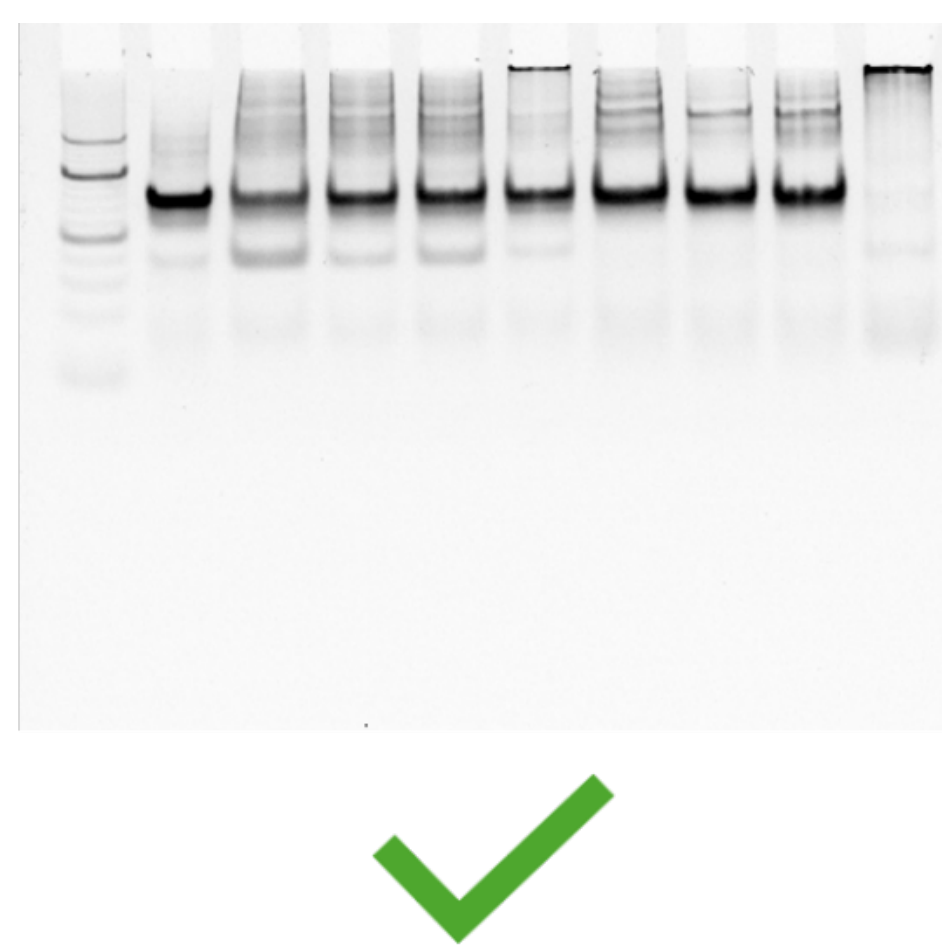

d. Click “Image Tools” in the Analysis Tool Box located on the left side of the program. Use the custom rotation tool to make the gel image level.

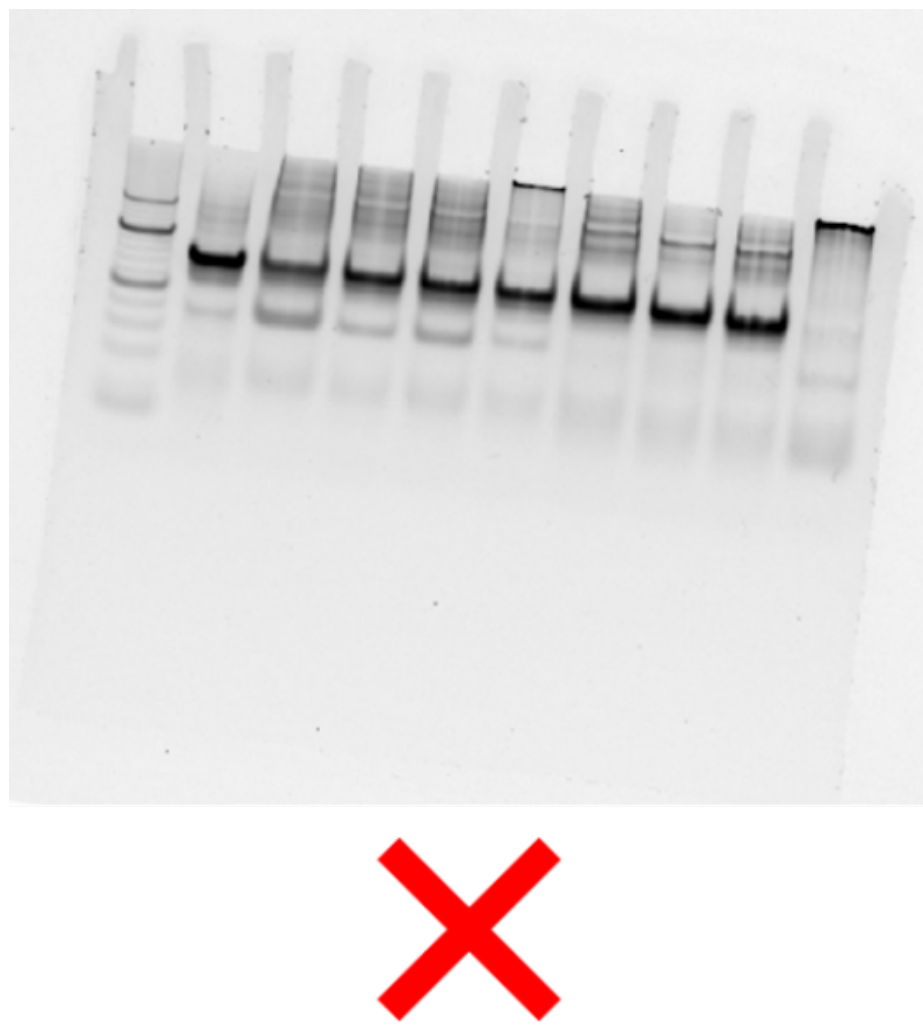

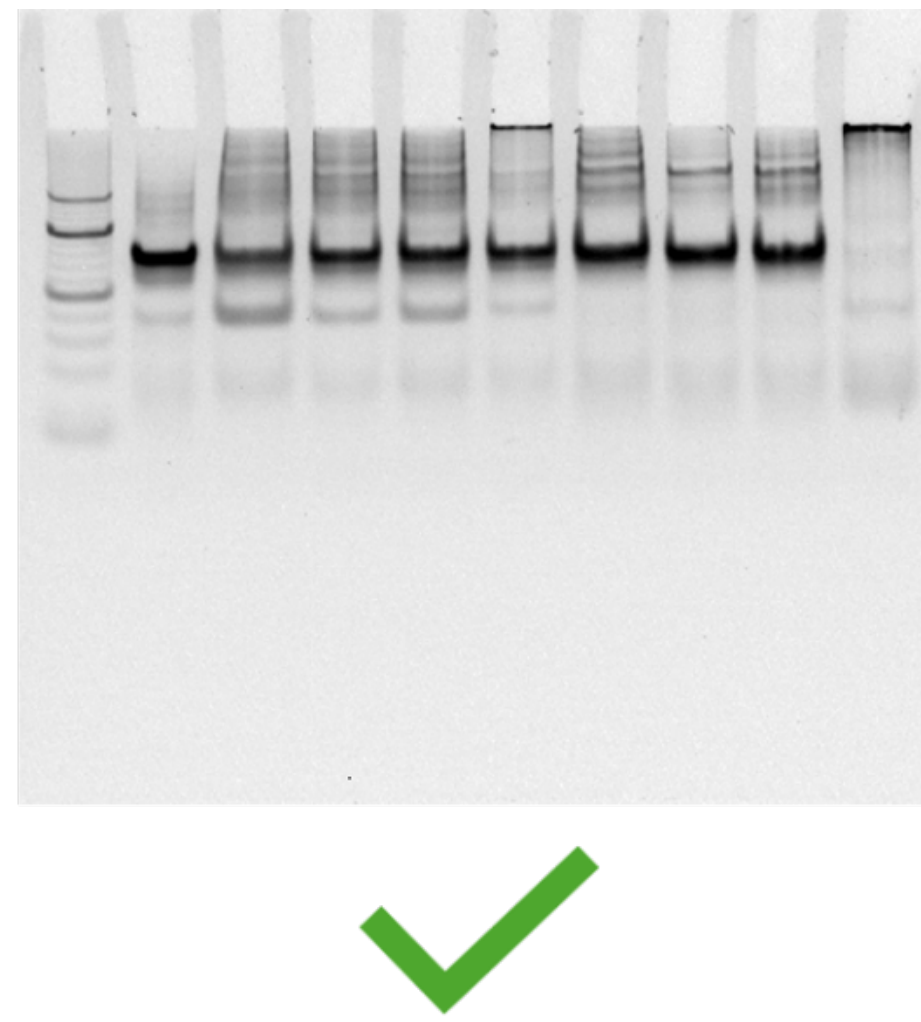

e. Click “Lane and Bands” in the Analysis Tool Box. In the “Lane Finder” box click “Automatic”. A blue box will appear around each of the lanes.

- You may need to adjust the placement of the lanes. Click and drag the lane outline to make sure it correctly aligns with the lanes of your gel.
- The ladder does not need to be included as it is not used for analysis.

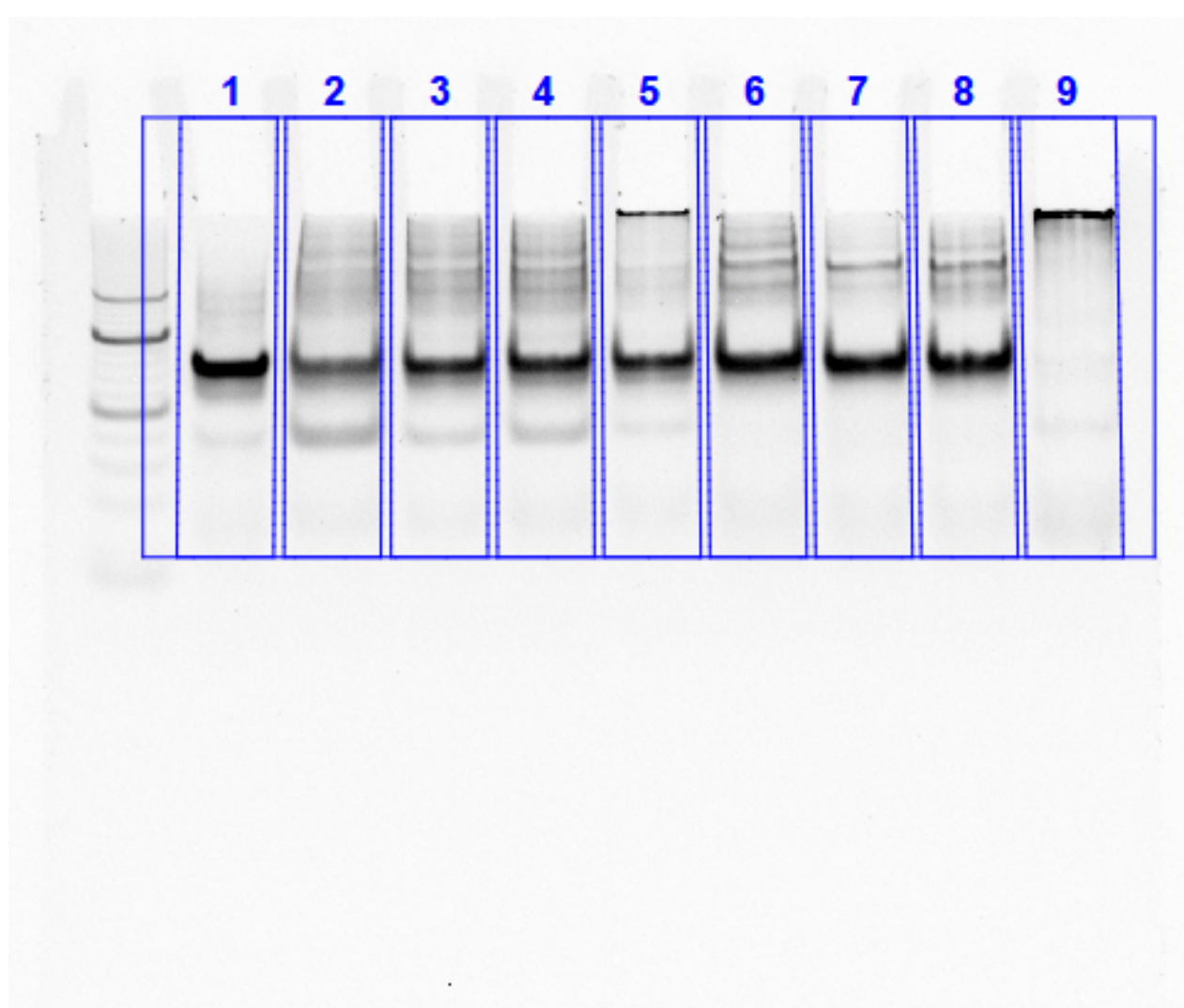

f. Click on “Bands” at the top of the “Lanes and Bands” section. In the “Bands” box, click “Add”. On the gel image, click on each of the bands corresponding to the DX motif in each of the lanes.

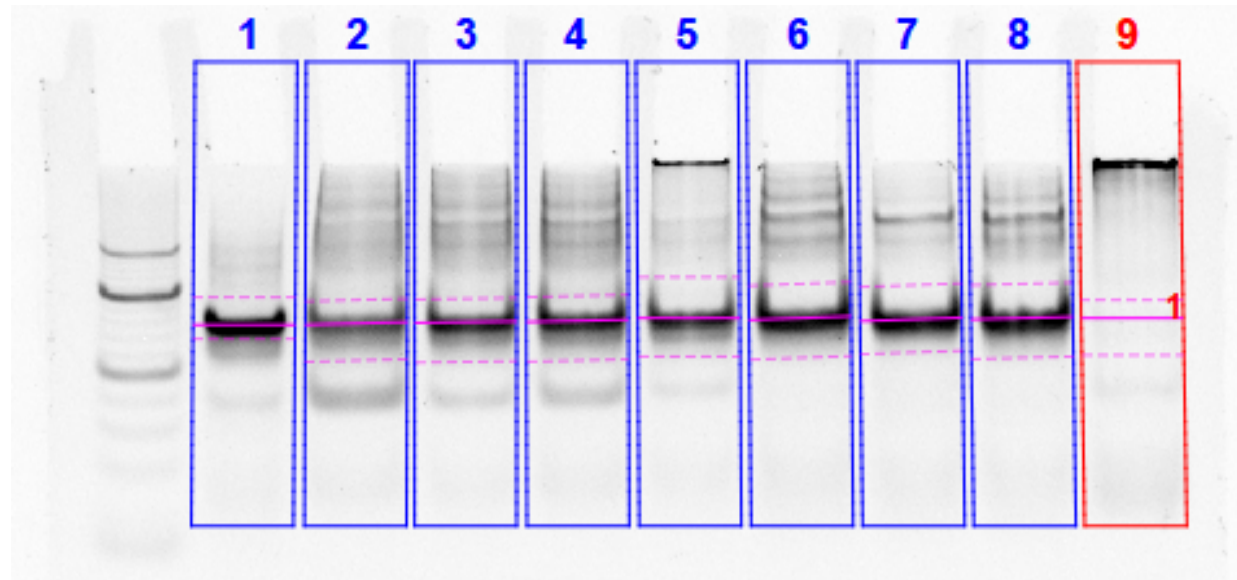


g. In the “Bands” box, click “Adjust”. Click and drag the pink dotted lines in the gel image to line them up with the top and bottom of each band.

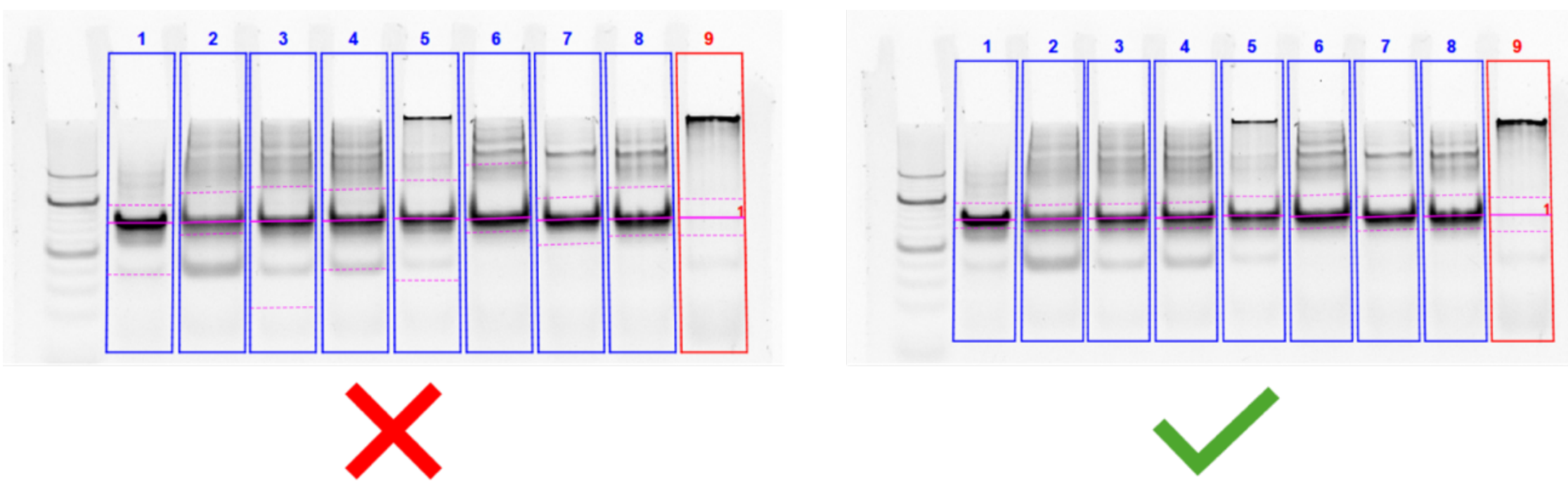


## 2. Normalizing yields of isothermal assemblies to control

a. Click “Analysis Table”, a new tab should open. Click the 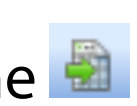 icon. This will open an Excel File with the data from the gel.

| Lane | Band No. | Band Labe | Mol. Wt. (K | Relative Fr | Adj. Volum | Volume (In | Abs. Quan | Rel. Quant | Band % | Lane % |
|---|---|---|---|---|---|---|---|---|---|---|
| 1 | 1 | | N/A | 0.566474 | 3850236 | 4192996 | N/A | N/A | 100 | 71.22311 |
| 2 | 1 | | N/A | 0.563584 | 2764500 | 2980492 | N/A | N/A | 100 | 38.8842 |
| 3 | 1 | | N/A | 0.560694 | 3954204 | 4511436 | N/A | N/A | 100 | 58.43001 |
| 4 | 1 | | N/A | 0.557803 | 4061212 | 4446988 | N/A | N/A | 100 | 54.36667 |
| 5 | 1 | | N/A | 0.552023 | 3426232 | 3884664 | N/A | N/A | 100 | 62.60259 |
| 6 | 1 | | N/A | 0.552023 | 4444556 | 4768772 | N/A | N/A | 100 | 61.08059 |
| 7 | 1 | | N/A | 0.554913 | 3365812 | 3570100 | N/A | N/A | 100 | 59.08794 |
| 8 | 1 | | N/A | 0.552023 | 3550644 | 3750676 | N/A | N/A | 100 | 53.1121 |
| 9 | 1 | | N/A | 0.549133 | 341772 | 465956 | N/A | N/A | 100 | 7.629534 |

b. For this analysis, we only need to use the Volume. Copy and paste this column into another section of the spreadsheet. Label each section with the contents of the lane.

| Sample | Volume (Int) |
|---|---|
| $Mg^{2+}$, annealed | 4192996 |
| $Mg^{2+}$, 20 °C | 2980492 |
| $Ca^{2+}$, 20 °C | 4511436 |
| $Sr^{2+}$, 20 °C | 4446988 |
| $Ni^{2+}$, 20 °C | 3884664 |
| $Mg^{2+}$, 50 °C | 4768772 |
| $Ca^{2+}$, 50 °C | 3570100 |
| $Sr^{2+}$, 50 °C | 3750676 |
| $Ni^{2+}$, 50 °C | 465956 |

c. Normalize the yields from each lane to that of the control. Divide each lane by the volume corresponding to the control.

| Sample | Volume (Int) | Normalized |
|---|---|---|
| $Mg^{2+}$, annealed | 4192996 | 1 |
| $Mg^{2+}$, 20 °C | 2980492 | 0.71082634 |
| $Ca^{2+}$, 20 °C | 4511436 | 1.075945696 |
| $Sr^{2+}$, 20 °C | 4446988 | 1.060575302 |
| $Ni^{2+}$, 20 °C | 3884664 | 0.926464991 |
| $Mg^{2+}$, 50 °C | 4768772 | 1.137318519 |
| $Ca^{2+}$, 50 °C | 3570100 | 0.851443693 |
| $Sr^{2+}$, 50 °C | 3750676 | 0.894509797 |
| $Ni^{2+}$, 50 °C | 465956 | 0.111127223 |

d. Plot the data as a bar graph according to guidelines provided by the instructor.

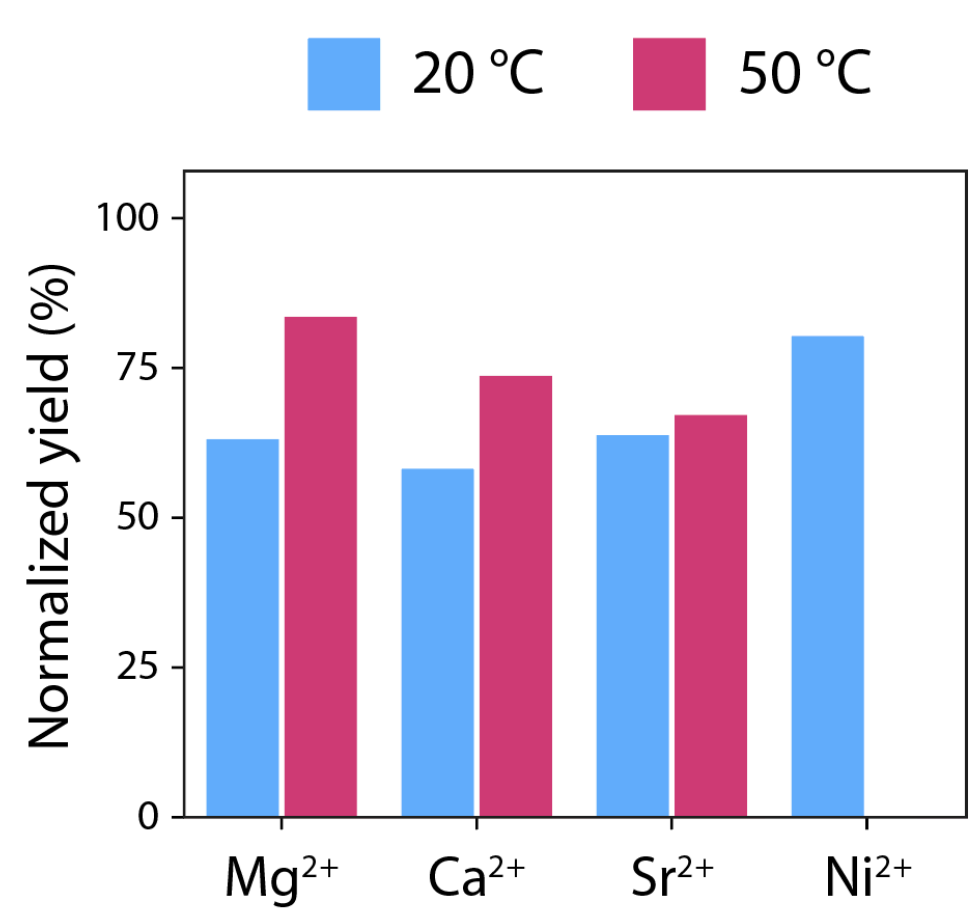

## Supplementary note 4: Troubleshooting gel electrophoresis

| Problem | Troubleshooting |
|---|---|
| Gel doesn't solidify | • Make sure to prepare the stocks fresh and accurately weigh all chemicals.<br>• Use freshly made 10% ammonium persulfate.<br>• After pouring gel solution in the casting cassettes, leave the remaining gel solution in the conical flask to check polymerization after some time. |
| Error appears in the gel power supply | • Make sure the buffer is filled to the marked lines on the chamber according to the number of gels used.<br>• Check if the wires are plugged into the power supply correctly (red to red and black to black). |
| Leakage of gel solution | • Pour gel solution steadily and stop immediately upon observing leakage. Use a spare casting kit and decant the gel solution in the spare kit instead.<br>• Check the casting kit and make sure the bottom is aligned with the bottom flap to avoid leaks. |
| Gel bands did not run evenly | • Check that gel gasket is not leaking prior and during the gel run time.<br>• Make sure gel plates are properly seated in gel gasket. |
| DNA samples are not present on gel image | • Ensure all necessary components were added during the assembly process.<br>• Restain and reimage the gel in case there was an error during the staining process.<br>• Make sure electrodes are plugged in properly (red to red, black to black) as the gel will run in reverse if electrodes are swapped. |

## Supplementary note 5: Stepwise assembly of DX motif

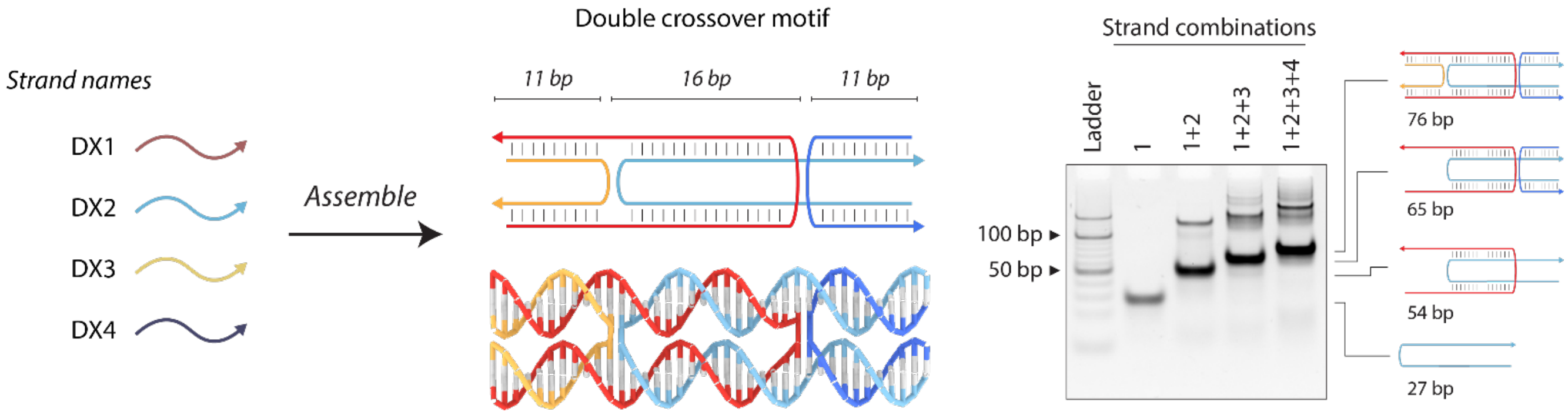


### Procedure

Follow the below procedure step-by-step to prepare the DNA double crossover (DX) motif for further understanding of the partial assembly of the motif.

### 1. Assembly of DX DNA motif in ion solutions [Timing: 60 min]

a. Prepare the DX DNA motif using single strands DX1, DX2, DX3 and DX4. 5 µM dilutions of each strand will be provided to you by your instructor.

b. Mix DNA samples with buffer following the table below. The samples are mixed in PCR tubes (or 0.5 ml or 1 ml tubes). You will prepare samples corresponding to partial assemblies from 1 oe 2 or 3 strands, and the full structure containing 4 strands (total 4 samples). Final sample volume is 10 µl.

| Reagent | Starting concentration | Final concentration | DX 1 | DX 1+2 | DX 1+2+3 | DX 1+2+3+4 |
|---|---|---|---|---|---|---|
| Strand DX1 | 5 µM | 0.5 µM | 1 µl | 1 µl | 1 µl | 1 µl |
| Strand DX2 | 5 µM | 0.5 µM | | 1 µl | 1 µl | 1 µl |
| Strand DX3 | 5 µM | 0.5 µM | | | 1 µl | 1 µl |
| Strand DX4 | 5 µM | 0.5 µM | | | | 1 µl |
| TAE with $Mg^{2+}$ | 10× | 1× | 1 µl | 1 µl | 1 µl | 1 µl |
| De-ionized water | | | 8 µl | 7 µl | 6 µl | 5 µl |
| Total volume | | | 10 µl | 10 µl | 10 µl | 10 µl |

c. Incubate the samples for 45 minutes at 50 °C in a thermal cycler to maintain temperature throughout the experiment.

Alternate: Mix samples in 0.5 ml or 1 ml tubes and keep on heating blocks set to 50 °C.

## 2. Preparing native polyacrylamide gel [Timing: 15 min]

a. Set up the gel plates and chamber according to instructor's directions.

b. Mix the following to make an 8% polyacrylamide gel.
   2 ml 40% polyacrylamide
   1 ml 10× TAE/$Mg^{2+}$
   7 ml de-ionized water

   Note: Add the APS and TEMED just before the gel is ready to be poured into the gel plate set-up.
   80 μl 10% APS
   4 μl TEMED

c. Pour the gel solution in the glass plate set up using a 10 ml serological pipet. Place the comb and allow gels to polymerize for 30-40 minutes.

d. Set up gel chamber and fill with 1× TAE-$Mg^{2+}$ for gel running.

e. Rinse wells by flushing running buffer using a 200 μl pipette.

## 3. Gel electrophoresis of DX samples [Timing: 60 min]

a. Add 1 μl loading dye to all the sample tubes.

b. Starting with the ladder provided by the instructor, load 10 μl of the ladder, and each of the samples into the gel, totaling 5 lanes.

   Note: Follow this loading order for the samples, from left to right.
   Ladder
   DX1
   DX1+DX2
   DX1+DX2+DX3
   DX1+DX2+DX3+DX4

c. Run the gel by applying 100 V for 45 minutes at room temperature.

   Note: Make sure to plug in the electrode to the matching sockets (red to red and black to black).

### 4. Gel staining and imaging [Timing: 30 min]

a. Prepare gel staining trays with 50 ml of water and add 5 µl of GelRed stock solution to the water and stir well using the pipette tip.

b. Open the gel plates and place the gel carefully in the staining solution. For convenience, gel can remain on one of the plates so that it is easier to move the gel.

c. Place the gel-containing trays on a rotator or a shaker, cover it with aluminum foil and let it stain for 10 minutes.

d. After staining, move the gel to a tray with 50 ml water. De-stain for 5 minutes.

e. Remove the gel from the de-staining tray and place on the imaging platform (without the gel plate) on a gel imager. Image using the gel analysis software. Make sure to take gel images at an exposure time at which the band corresponding to the structure is not saturated. If saturated, the intensity values will not be accurate. The exposure time in the imager can be reduced to obtain gel images without saturation of the bands.
